# Supplementary material for: The causal relationship between the human gut microbiota and pyogenic arthritis: a Mendelian randomization study
Source: Front Cell Infect Microbiol. 2024 Nov 26;14:1452480. doi: 10.3389/fcimb.2024.1452480 (PMC11629706; doi:10.3389/fcimb.2024.1452480)
Supplement: Supplementary file 1 [file DataSheet1.zip › mendelian test/ebi-a-GCST90027596.csv_leave_one_out.pdf]

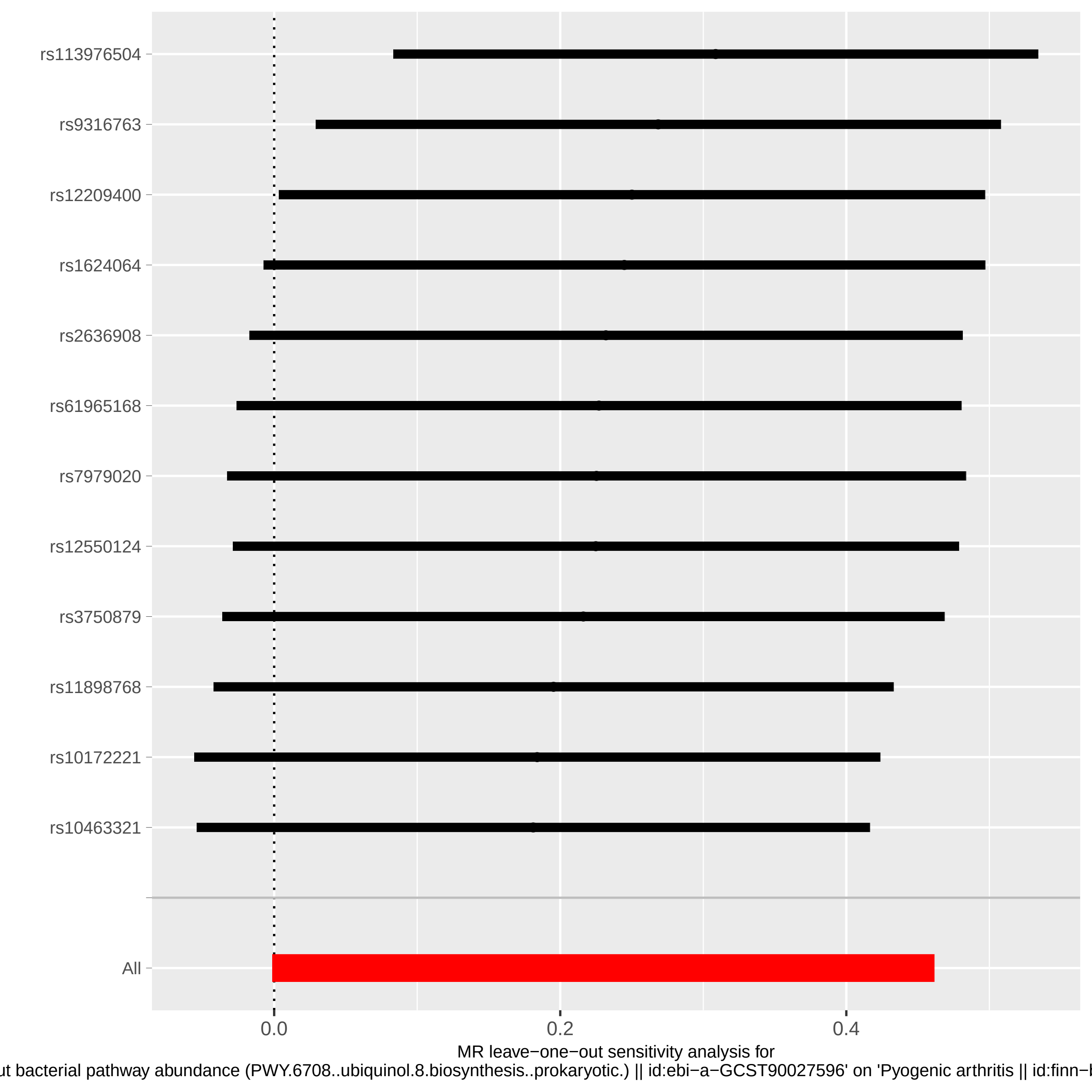
rs113976504
rs9316763
rs12209400
rs1624064
rs2636908
rs61965168
rs7979020
rs12550124
rs3750879
rs11898768
rs10172221
rs10463321
All
0.0
0.2
0.4
MR leave−one−out sensitivity analysis for
ut bacterial pathway abundance (PWY.6708..ubiquinol.8.biosynthesis..prokaryotic.) || id:ebi−a−GCST90027596' on 'Pyogenic arthritis || id:finn−
